# Angiogenesis-related genes may be a more important factor than matrix metalloproteinases in bronchopulmonary dysplasia development

**DOI:** 10.18632/oncotarget.14722

**Published:** 2017-01-18

**Authors:** Min Yang, Bo-Lin Chen, Jian-Bao Huang, Yan-Ni Meng, Xiao-Jun Duan, Lu Chen, Lin-Rui Li, Yan-Ping Chen

**Affiliations:** ^1^ Respiratory Department 2, Hunan Children’s Hospital, Changsha, Hunan, China; ^2^ Thoracic Medicine Department 2, Hunan Cancer Hospital, Changsha, Hunan, China

**Keywords:** bronchopulmonary dysplasia, chronic lung disease, premature infant, THBS1, MMPs, Pathology Section

## Abstract

We characterized the expression profile of angiogenesis-related genes (ARG) and matrix metalloproteinase (MMP) genes in preterm infants, with and without bronchopulmonary dysplasia (BPD). We reanalyzed a gene expression dataset for preterm infants from the Gene Expression Omnibus database using the Gene-Cloud of Biotechnology Information platform. A total of 1,652 genes were differentially (1.2-fold change) expressed: 811 were highly expressed in infants with BPD, and 841 were highly expressed in those without BPD. Twenty-eight and 11 ARGs were upregulated in infants with and without BPD, respectively. Among 27 detected MMPs and TIMPs, MMP8, MMP9, MMP25, TIMP2 and TIMP3 were differently expressed. Levels of THBS1, MMP8, MMP9, MMP25, TIMP2 and TIMP3 increased as severity of BPD and retinopathy of prematurity (ROP) increased, whereas ETS1, LEF1 and SPOCK2 exhibited the opposite trend. Expression of ETS1 and LEF1 had a fitting rate of *R*^2^ = 0.849 and *P* < 0.001. ELISAs showed a positive correlation between THBS1 and CD36 (receptor of THBS1) levels in serum samples from preterm infants. Our study indicates that the upregulation of THBS1 and downregulation of ETS1, LEF1 promotes BPD in preterm infants by disrupting blood vessel formation rather than by dysregulation of MMPs and TIMPs.

## INTRODUCTION

Bronchopulmonary dysplasia (BPD) is the most common chronic respiratory disease in premature infants and causes significant morbidity and mortality [1–3]. BPD results from antenatal and perinatal factors that interrupt lung development in infants born at the extremes of prematurity [4, 5]. Minimizing environmental stressors is insufficient to prevent BPD or to decrease its incidence [5, 6]. Next generation sequencing and other techniques show that BPD is a consequence of disruption of normal pulmonary vascular and alveolar growth. Growth factors, cytokines, signaling molecules and transcription factors and inflammatory mediators can influence alveologenesis and vascularization [7]. In addition to arrested alveolar development, impaired pulmonary microvascular development occurs in infants with BPD [8–10] and in BPD-like animal models [11, 12]. Matrix metalloproteinases (MMPs) are also a factor in digesting and degrading extracellular matrix (ECM) during lung tissue remodeling and BPD development [13–15].

Although angiogenesis and MMP genes inhibit alveologenesis or microvascular development in BPD, the molecular mechanism remains undiscovered. A gene expression dataset, developed by Pietrzyk et al [7] and uploaded to the Gene Expression Omnibus (GEO) database (GSE32472 http://www.ncbi.nlm.nih.gov/geo/query/acc.cgi?acc = GSE32472), was reanalyzed. The study included 111 newborns, 68 of which were BPD preterm infants and 43 of which were control subjects. Three blood samples were collected from each participant newborn on the 5th, 14th and 28th day of life. The mRNA samples were evaluated for gene expression by use of GeneChip^®^ Human Gene 1.0 ST microarrays. The whole-genome expression study revealed alteration of the expression of nearly 10% of the genome in bronchopulmonary dysplasia patients [7]. By reanalyzing this dataset, we illustrated the relation between BPD and the expression profile of angiogenesis-related genes (ARGs) as well as MMP genes.

## RESULTS

### Cluster analysis of GSE32472 and 39 ARGs

**Figure 1:**
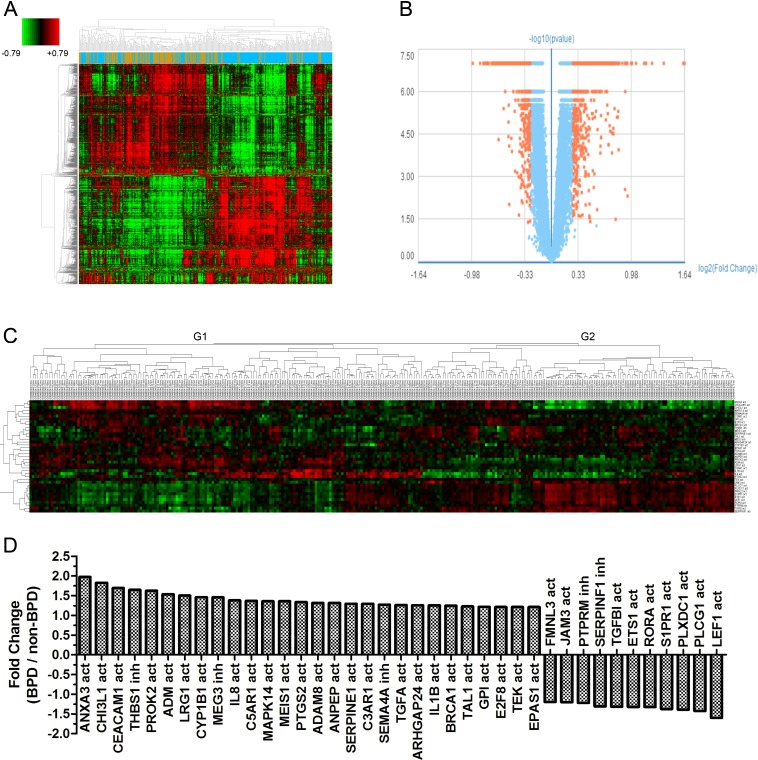
Graphs show ARGs expression in preterm infants **A**. Heatmap shows differential expression genes in 294 samples (Brown: no-BPD infant samples, 112; Blue: BPD infant samples, 182). **B**. Volcano plot shows differential expression genes in BPD and no-BPD preterm infants (Fold change > 1.2, *P* < 0.05). **C**. Cluster analysis for 39 detected ARGs in 294 samples. (no = no-BPD; mi = mild BPD; mo = moderate BPD; se = severe BPD; act = activation; inh = inhibition) **D**. Fold change of 39 ARGs in BPD and no-BPD preterm infants.

Gene expression data of 299 samples were obtained from the GSE32472 dataset. For 5 samples, disease classification information was missing, so the total number of samples in the reanalysis was 294. Demographic and clinical characteristics of the neonates are show in Table 1. In BPD and no-BPD preterm infants, 1,652 genes were considered as different expression genes (DEGs). Figure 1A and 1B show the DEGs in BPD and no BPD samples. ARG gene symbols were obtained from AmiGO2 (http://amigo.geneontology.org/amigo/landing) and gene ontology (GO) analysis results and then identified as activators or inhibitors of angiogenesis. In BPD and no-BPD samples, 39 ARGs were differently expressed. Cluster analysis shows that ARGs classified the samples into two groups (Figure 1C). Group 1 (G1) consisted of 42 no-BPD samples and 64 mild, 25 moderate and 33 severe BPD samples. Group 2 (G2) consisted of 70 no-BPD samples and 42 mild, 9 moderate and 9 severe BPD samples. The χ^2^ test showed a significantly different component ratio between G1 and G2 (Figure S1). The number of upregulated ARGs in G1 and G2 was 27 and 12, respectively. The genes highly expressed in G1 and G2 were just the genes highly expressed in the no-BPD group and the BPD group, respectively (Figure 1D).

**Table 1:** Demographic and clinical characteristic of premature infants

	BPD	No BPD	*P*
Birth weight, g (mean ± SD)	882.42 ± 29.23	1235.66 ± 37.34	<0.001^1^
gestational age (weeks) (mean ± SD)	26.37 ± 0.24	29.74 ± 0.3	<0.001^1^
Female gender	23 (37.1%)	21 (55.26%)	0.0757^2^
Severity of BPD^3^			
No BPD	0	40	
Mild	38	0	
Moderate	13	0	
Severe	15	0	

^1^*p*-value for *t*-test.

^2^*p*-value for χ^2^ test.

^3^ The number of each classification of BPD sample is a little bit different from the original article of the dataset.

Cluster analysis showed the expression of 1,652 DEGs and 39 ARGs on each single day (5th, 14th and 28th) could also approximately divide the samples into two groups, G1 and G2 (Figure S2 – Figure S5, Table S1–Table S4).

**Figure 2:**
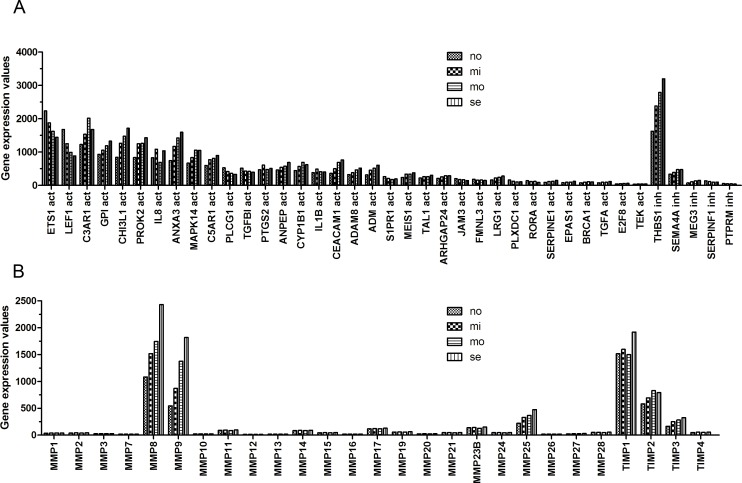
The expression of ARGs, MMPs and TIMPs at different severity levels of BPD samples **A**. The expression of 39 ARGs. THBS1 showed high expression activity. **B**. MMP8, MMP9 and TIMP1 exhibited high expression value.

### ARGs, MMPs and TIMPs expression in preterm infant samples

The expression values of ARGs, MMPs and tissue inhibitor of metalloproteinases (TIMPs) at different severity of the disease were obtained (Figure 2). Among 39 ARGs, 28 (71.79%) were upregulated and 11 (28.21%) were downregulated in BPD samples. Thirty-four ARGs were angiogenic genes, 25 (73.53%) were upregulated and 9 (26.47%) were downregulated in BPD samples. The other 5 genes were antiangiogenic. THBS1 is one of the anti-angiogenic genes that expressed actively in preterm infants. The more severe the BPD disease, the higher the expression level of THBS1. Two of the angiogenic genes, ETS1 LEF1, were also expressed actively but showed a different trend than THBS1 and most of the other angiogenic genes (Figure 2A).

Among 27 detected MMPs and TIMPs, MMP8, MMP9, MMP25, TIMP2 and TIMP3 were differently expressed. MMP8, MMP9 and MMP25 were highly expressed in BPD infants. The expression of these genes increased with the severity of BPD. TIMP1 and TIMP2 were also expressed actively in preterm infants.

### The expression of ARGs, MMPs and TIMPs with different severity of BPD, ROP and gestational age

**Figure 3:**
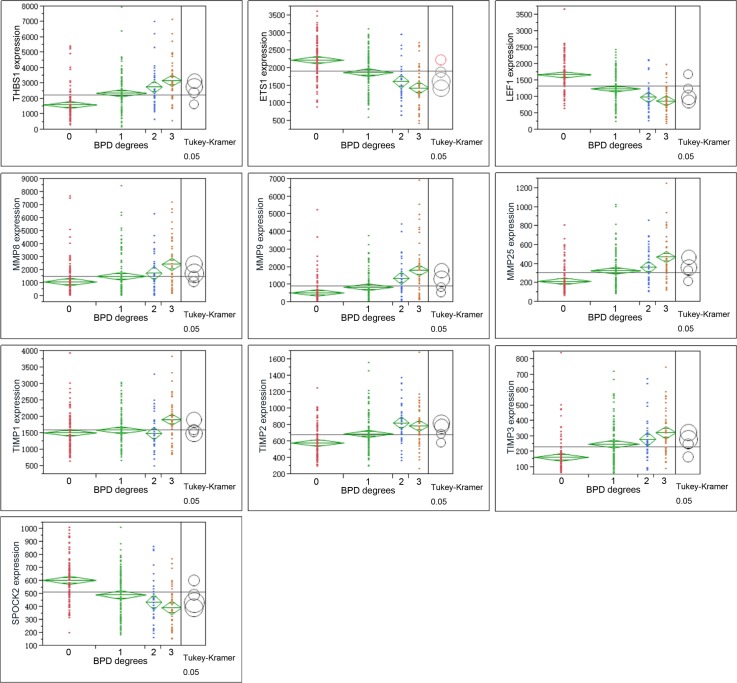
Graphs show ANOVA of gene expression of THBS1, LEF1, ETS1, MMPS and TIMPs at different severity levels of BPD samples Multiple comparisons were done by Tukey-Kramer, *P* < 0.05. Circles for means that are significantly different either do not intersect or intersect slightly, so that the outside angle of intersection is less than 90 degrees. If the circles intersect by an angle of more than 90 degrees, or if they are nested, the means are not significantly different. For BPD degree, 0 = no-BPD, 1 = mild BPD, 2 = moderate BPD, 3 = severe BPD.

**Figure 4:**
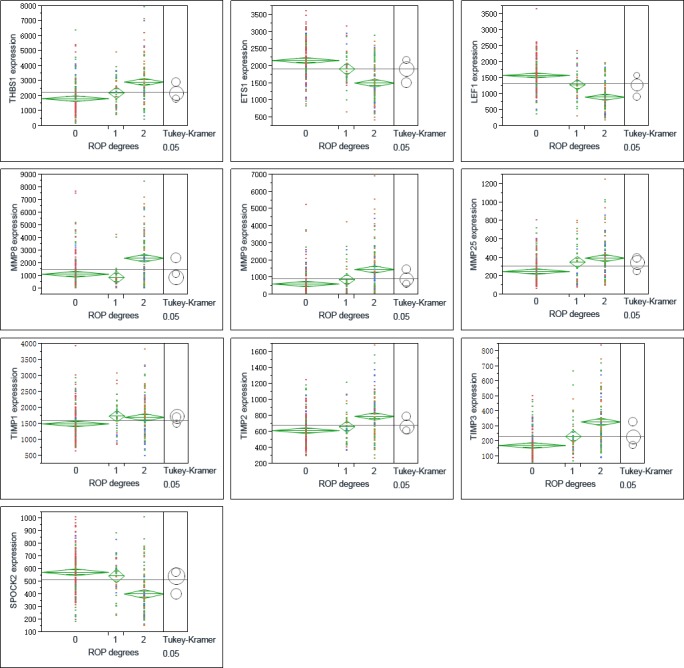
Graphs show ANOVA of gene expression of THBS1, LEF1, ETS1, MMPS and TIMPs at different severity levels of ROP Multiple comparisons were done by Tukey-Kramer, *P* < 0.05. 1 = no ROP, 2 = ROP not requiring treatment, 3 = ROP requiring laser therapy.

**Figure 5:**
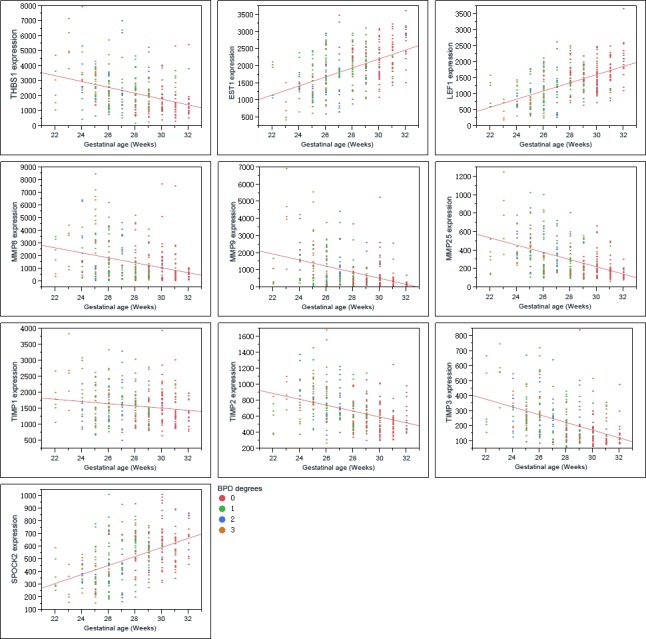
Graphs show the correlations of the genes as indicated with birth gestational age of the preterm infants

One-way analysis of variance (ANOVA) was performed on ARGs, MMPs and TIMPs for different severity of BPD (Figure 3). The relations of these genes to retinopathy of prematurity (ROP) and gestational age of the infants were also observed (Figure 4, Figure 5).

THBS1 expression increased along with the severity of BPD, which showed a different trend with LEF1, ETS1 (Figure 3, Figure 4). ROP is a retinal vascular disease in premature infants that is caused by incomplete vascularization and abnormal fibrovascular proliferation. We analyzed the gene expression of ARGs, MMPs and TIMPs for different levels of ROP and the relation of these genes to gestational age. THBS1 showed no significant difference between the no ROP and the ROP not requiring treatment groups. The expression of THBS1, MMPs and TIMPs was lower with increased gestational age of the infants. The expression of ETS1, LEF1 was higher with increased weeks of age (Figure 5).

Hadchouel et al [1] conducted a genome-wide association study in two different ethnic populations. They found that the SPOCK2 gene was the only BPD-associated gene that emerged in their analyses. In addition to this finding, mRNA studies showed that SPOCK2 was expressed in the developing lung and SPOCK2 mRNA in whole-lung tissue increased gradually after birth [1]. SPOCK2 expression in our reanalysis showed that the downregulation of SPOCK2 was associated with BPD and ROP levels, and the expression of SPOCK2 increased as the gestational age of the infants increased (Figure 3 Figure 5).

Linear fit results in Figure 5 show the relation between the genes as indicated and the gestational age of the infants. The expression of upregulated genes in BPD samples was lower with increased gestational age, whereas downregulated genes in the BPD samples showed a different trend. Because the gene expression was associated both with the BPD level and with birth gestational age, we performed a scatterplot 3D analysis of these three factors. Figure S7 shows the younger the infants, the more severe the disease and the higher the THBS1 expression. The same trend was found in MMP8, MMP9, MMP25 and TIMPs, whereas LEF1, ETS1 and SPOCK2 exhibited a different trend.

### Multivariate analysis shows the correlation of the ARGs, MMPs and TIMPs

Angiogenesis is controlled by the net balance between molecules that have positive and negative regulatory activity [16]. ARGs, MMPs, and TIMPs are important factors in BPD development [8–10, 13–15, 17], and the synergistic action and balance of ARGs, MMPs and TIMPs are important during angiogenesis [18]. We found that some MMPs, TIMPs and ARGs existed the same expression trend at different severity of BPDs. Multivariate analysis was performed and correlation coefficient (R's square value) of the genes was calculated and that LEF1 and ETS1 exhibited good correlation (*r* = 0.9213, *P* < 0.001), as shown in Figure S8. The rest correlation coefficients were shown in supplementary.

### ROC analysis for pathological discrimination

Receiver operating characteristic curves (ROC) analysis was performed and area under the curve (AUC) of ARGs, MMPs, and TIMPs was obtained (Figure S9). According to area under the curve (AUC) of all variables, ARGs had a better differential capability than MMPs and TIMPs.

### The protein levels of ARGs, MMPs and TIMPs

To verify the microarray data, ELISA experiments were performed. Serum samples were obtained from the Hunan Children's Hospital. The demographic and clinical characteristics of premature infants are shown in the Table 3.

**Table 2:** ARGs from AmiGO2 and GO analysis

GO ID	GO Name	Diff / Total Gene in GO	*p*-value	Gene Symbols
GO:0001525	angiogenesis	20/201	1.07E-05	IL8 PROK2 PTGS2 CYP1B1 GPI PLXDC1 TGFBI ARHGAP24 JAM3 TEK SERPINE1 CEACAM1 TAL1 MEIS1 MAPK14 S1PR1 ANPEP ADAM8 EPAS1 TGFA
GO:0045766	positive regulation of angiogenesis	13/84	8.34E-06	ANXA3 ADM PLCG1 ETS1 TEK CHI3L1 SERPINE1 CYP1B1 C3AR1 IL1B BRCA1 C5AR1 LRG1
GO:0002040	sprouting angiogenesis	2	—	E2F8 LEF1
GO:0016525	negative regulation of angiogenesis	5	—	THBS1 SEMA4A MEG3 SERPINF1 PTPRM

**Table 3:** Demographic and clinical characteristic of premature infants from Hunan Children's Hospital

	BPD	No-BPD	*P*
Birth weight, g (mean ± SD)	1110±425	1121±432	>0.05^1^
gestational age (weeks) (mean ± SD)	30.7±2.5	30.8±2.7	>0.05^1^
Female gender	15 (15/45)	10 (10/32)	0.8473^2^

^1^*p*-value for *t*-test.

^2^*p*-value for χ^2^ test.

**Figure 6:**
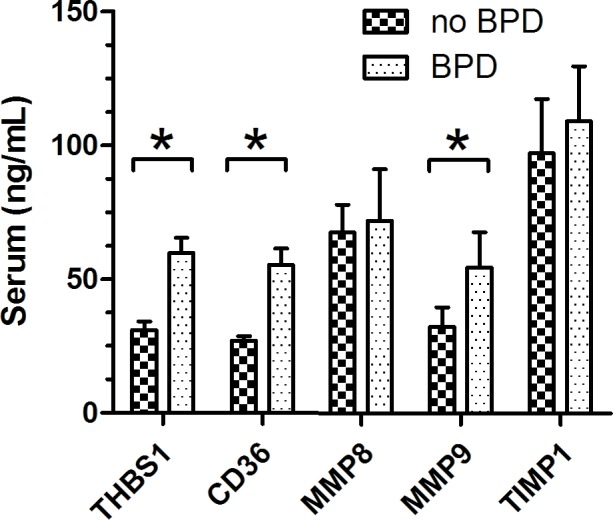
ELISA experiments show the levels of THBS1, CD36, MMP8 and MMP9 THBS1, CD36, and MMP9 were differently expressed in no-BPD and BPD samples, *P* < 0.05. For no-BPD, *n* = 32; for BPD, *n* = 45.

ELISA experiments were performed per the protocol of the kits. Figure 6 shows the protein levels of THBS1, CD36 and MMP9 were significantly different between BPD and no-BPD samples. ETS1, LEF1, MMP25, TIMP2 and TIMP3 were not detected in our serum samples. CD36, a receptor of THBS1, was also detected by ELISA.

## DISCUSSION

BPD is a complex disease with a strong genetic component. Some researchers believe a number of genetic variants and pathways could be associated with the susceptibility of BPD. Each genetic variant likely contributes to the development of the disease, and together these variants cause dysregulation in biological pathways in the developing lungs of premature infants [19]. Preterm birth causes impaired vascular and alveolar growth that leads to BPD. A better understanding of the mechanisms of impaired pulmonary vascular growth will lead to novel therapies for this high-risk population [4].

We checked the expression profile of ARGs (Figure 1). ARGs divided the samples into two groups (G1 and G2). In addition, among 34 angiogenic genes, 25 (73.53%) were upregulated and 9 (26.47%) were downregulated in BPD samples. The data suggested that ARGs were associated with BPD. We observed the relation between the levels of the genes and the severity of the disease (Figures 2–3). Figure 2A and Figure 3 show the anti-angiogenic gene THBS1 and the angiogenic genes ETS1, LEF1 were significantly upregulated and downregulated, respectively in BPD samples. We propose that ETS1, LEF1 and THBS1 may participate in BPD development by disturbing the formation of blood vessels.

Both *in vitro* and *in vivo* data show that the main function of THBS1 is as a potent inhibitor of angiogenesis, modulating angiogenesis by acting on endothelial cells and vascular smooth muscle cells [20–22]. A microarray analysis conducted by De Paepe et al [10] found THBS1 to be one of the most overexpressed genes in ventilated lungs among 112 ARGs in ventilated preterm human lungs, which is consistent with our result (Figure 2A). In our analysis, THBS1 was extremely activated in preterm infants, and both microarray and ELISA results illustrated that the expression of THBS1 was upregulated in BPD samples. Evidence suggests that lung blood vessels actively promote alveolar growth during development and contribute to the maintenance of alveolar structures throughout postnatal life [23]. Thebaud and Abman [23] concluded that lung angiogenesis, *via* the secretion of angiogenic growth factors, contribute to normal alveolar development, and impaired alveolar development in BPD is associated with arrested and dysmorphic vascular growth and decreased lung angiogenic growth factor expression. Exogenous experiments suggested that angiogenic growth factors may have the function of therapy for preserving or enhancing alveolar structure.

Because the main function of THBS1 is inhibitor of angiogenesis, and the expression of THBS1 is associated with the severity of BPD, we propose that high expression of THBS1 in BPD samples is a cause of the disease rather than a reparative or epiphenomenal factor. We failed to detect the expression of ETS1 and LEF1 by ELISA, possibly because of the sensitivity of the ELISA kits we used or the concentrations of the proteins in the serum were too low. The expression of ETS1 and LEF1 exhibited the opposite trend against THBS1 (Figure 3 Figure 5), suggesting that THBS1 may be an inhibitory factor for the expression of these two genes. BPD is associated with the inhibition of angiogenesis [3, 4, 6, 10], however we found that 25 (73.53%) of angiogenic genes were upregulated in BPD infants, and only one antiangiogenic gene, THBS1, was extremely upregulated in the BPD samples. The upregulation of anti-angiogenesis gene THBS1 may be an important factor in BPD blood vessel development, and the upregulation of the angiogenic genes may be a reparative response of the inhibition of angiogenesis.

Apart from vascular genes, MMPs are an important factor in BPD development. MMPs form a family of enzymes that mediate the degradation of extracellular matrix [24]. MMP9 is capable of degrading collagen IV, which is a major component of the basement membrane of airways [25]. Besides, MMPs are also an important factor in angiogenesis [18].

We found both MMP9 mRNA and protein highly expressed in BPD samples. MMP8 and MMP25 showed high expression in BPD samples, and we found no significant difference in TIMPs levels between no-BPD and BPD samples. Although expression of MMP8 and MMP9 was associated with worse disease states [14, 15], we do not consider high expression of MMP8 and MMP9 as negative factors for BPD infants. Lukkarinen et al [13] showed that MMP9 deficiency worsens alveolar hypoplasia in a transgenic mice model of BPD. They propose that MMP9 activity in the newborn lung may be a host-defense mechanism that protects the lung against inflammatory injury [13]. According to Mizikova and Morty [17], starting from the 24th week of gestational age, at which point the lung enters the saccular stage, the expression of MMP9 consistently increasing in normal infants. We found that the MMP9 expression level decreased as the preterm infant gestational age increased. The younger the infant the higher the expression (Figure 5). Whether the abnormal expression of a molecule is a cause of the disease or a consequence of it is sometimes difficult to tell. A molecule may be epiphenomenal, causal or reparative. We propose that the dynamic regulation of MMP9 is interrupted by the premature birth, mechanical ventilation and the upregulation of MMP9 in preterm infants is a reparative factor for BPD infants rather than a cause or epiphenomenal of the disease.

Further studies such as the relations of THBS1 to LEF1 and EST1, the mechanism behind the correlation of LEF1 and EST1, should be done.

## CONCLUSIONS

We confirmed that gestational age is relevant for BPD progression (Figure S6), and we detected a few dysregulated genes in preterm BPD infants. We confirmed that THBS1 is an important factor in BPD development, and we discovered that the expression of ETS1 and LEF1 have a high R's square value of line regression. Our study suggests that the dysregulation of ARGs like THBS1, ETS1, LEF1 is an important factor in BPD development rather than MMPs and TIMPs.

## MATERIALS AND METHODS

### GEO data reanalysis

The GEO data GSE32472 (http://www.ncbi.nlm.nih.gov/geo/queryacc.cgi?acc = GSE 32472) was downloaded from the GEO database and reanalyzed by GCBI (https://www.gcbi.com.cn/). For DEGs, Gene ontology, pathway analysis and gene network analysis were performed. Heatmap, volcano plot, gene expression data and gene annotation were utilized for further analysis. Gestational age, weight, gender, severity of the disease and ROP status of the infants were downloaded from the GEO database. Details about the newborns can be obtained from Pietrzyk et al [7].

### Gene cluster for DEGs and ARGs

For DEGs, GO analysis and pathway analysis were performed. ARGs were obtained from the AimGO 2 and GO analysis results (GO name) and identified as stimulator or inhibitor of angiogenesis (Table 2). Then angiogenesis genes were analyzed and displayed using Cluster and TreeView software [26, 27]. DEGs and ARGs expression values on each single day (5^th^, 14^th^ and 28^th^ day of birth) were also displayed by Cluster and TreeView software. MMPs expression information was obtained.

### ELISA verification for microarray data

To verify the microarray results and check the expression of the proteins as indicated in preterm infants, the serum samples of BPD and no-BPD infants were obtained from the Hunan Children's Hospital. The informed consents were signed by the parents of the infants, and the research was permitted by the research ethics board of Hunan Children's Hospital. The entry criteria were (a) preterm birth < 32 weeks gestational age, (b) birth weight ≤ 1500 g and (c) the need for respiratory support. The diagnostic criteria were proposed by Jobe and Bancalari [3].

ELISA kits (Abcam) were used to measure the levels of ARGs, MMPs and TIMPs in preterm infant serum.

### Statistical analysis

The reanalysis methods of the GEO data were published on GCBI official website (http://college.gcbi.com.cn/help/index.jhtml). Values are showed as mean ± standard deviation (SD). The Student *t* test and the χ^2^ test were used to evaluate the statistical significance of demographic and clinical characteristics of BPD and no-BPD infants by JMP. ELISA results of BPD and no-BPD infants were compared by use of the two-tailed Student *t* test. The significance level was set at *P* < 0.05. ANOVA and multiple comparisons were performed to check the gene levels among different severities of the disease. Three-D scatterplot analysis was performed to observe the relations of the genes to BPD severity and gestational age. Multivariate analysis was performed and correlation coefficient (R's square value) of the genes was calculated. Receiver operating characteristic curves (ROC) of ARGs, MMPs, and TIMPs for differentiation between no-BPD and BPD samples were analyzed using JMP.

## SUPPLEMENTARY MATERIALS FIGURES AND TABLES






