# Supplementary material for: Angiogenesis-related genes may be a more important factor than matrix metalloproteinases in bronchopulmonary dysplasia development
Source: Oncotarget. 2017 Jan 18;8(12):18670–9. doi: 10.18632/oncotarget.14722 (PMC5386638; doi:10.18632/oncotarget.14722)
Supplement: Supplementary file 1 [file oncotarget-08-18670-s001.pdf]

Angiogenesis-related genes may be a more important factor than matrix metalloproteinases in bronchopulmonary dysplasia development

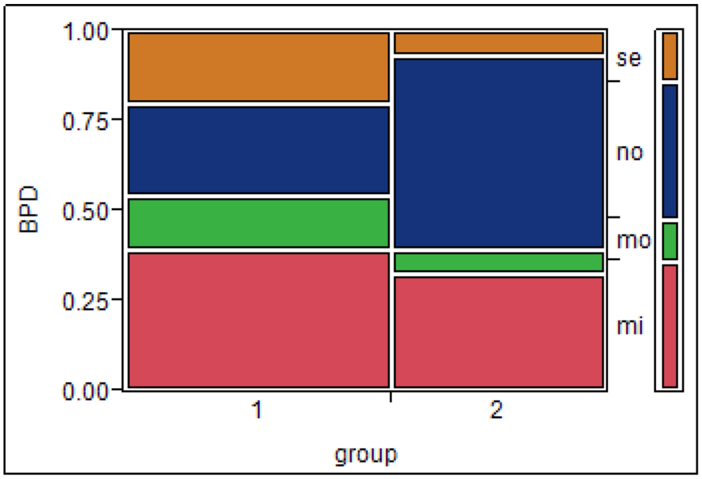

Tests

N	DF	-LogLike	RSquare (U)
294	3	15.074199	0.0406

Test	ChiSquare	Prob>ChiSq
Likelihood Ratio	30.148	<.0001*
Pearson	29.269	<.0001*

Supplementary Figure **S1**: The χ^2 test showed component ratio of different BPD levels for 294 samples divided by 39 ARGs. (no = no BPD, mi = mild BPD, mo = moderate BPD, se = severe BPD)

We observed the different expression gene profile on each single day (5th, 14th and 28th). As show in Figure S2 the samples on each single day can be approximately divided into two groups. Fisher's exact test shows the proportion of BPD levels in G1 and G2 were significantly different (Table S1 & S2).

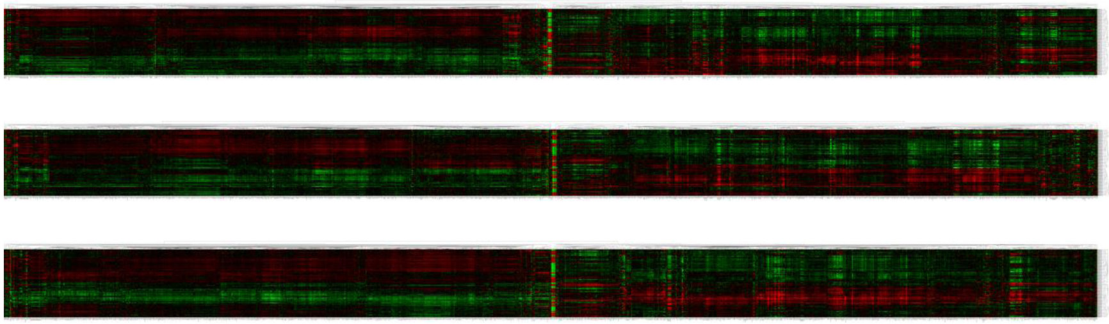

Supplementary Figure **S2**: Graphs show 1,652 DEGs on 5th, 14th, 28th day of birth divide the samples into two groups.

Supplementary Table **S1**: The proportion of BPD levels in G1 and G2 on each single day (5th, 14th, and 28th day of birth) (1652 DEGs)

BPD	5day		14day		28day	
degree	G1	G2	G1	G2	G1	G2
no	33	2	27	12	32	6
mi	27	17	18	16	11	27
mo	3	10	2	9	2	8
se	4	11	1	12	4	10
Total	57	40	48	49	49	51

Supplementary **Table S2**: Fisher's exact test for BPD and no-BPD counts in G1 and G2 by 5th, 14th, and 28th day samples (1652 DEGs)

	Group	no-BPD	BPD	P value
5th day	G1	33	24	< 0.01
	G2	2	38	
14th day	G1	27	21	< 0.01
	G2	12	37	
28th day	G1	32	17	< 0.01
	G2	6	45	

The cluster analysis shows that 39 angiogenesis-related genes (ARG) can approximately divide the samples, exsanguinated on 5th, 14th, 28th day of birth, into two groups (Fig.S3-Fig.S5, Tab.S2, and Tab.S3).

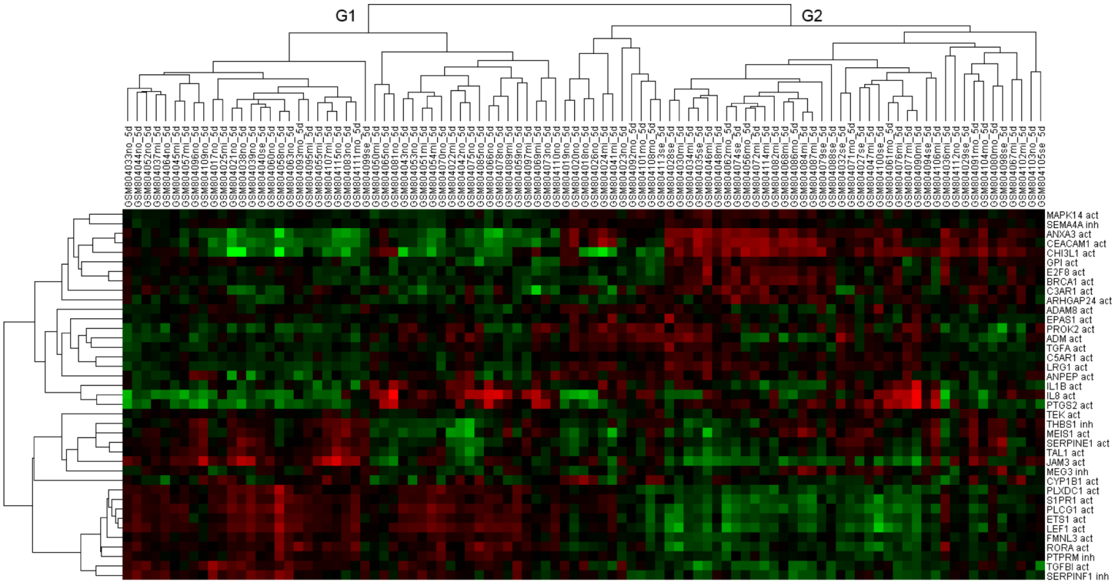

Supplementary Figure **S3**: Cluster analysis shows the 39 ARGs divide the samples of 5th day into two groups.

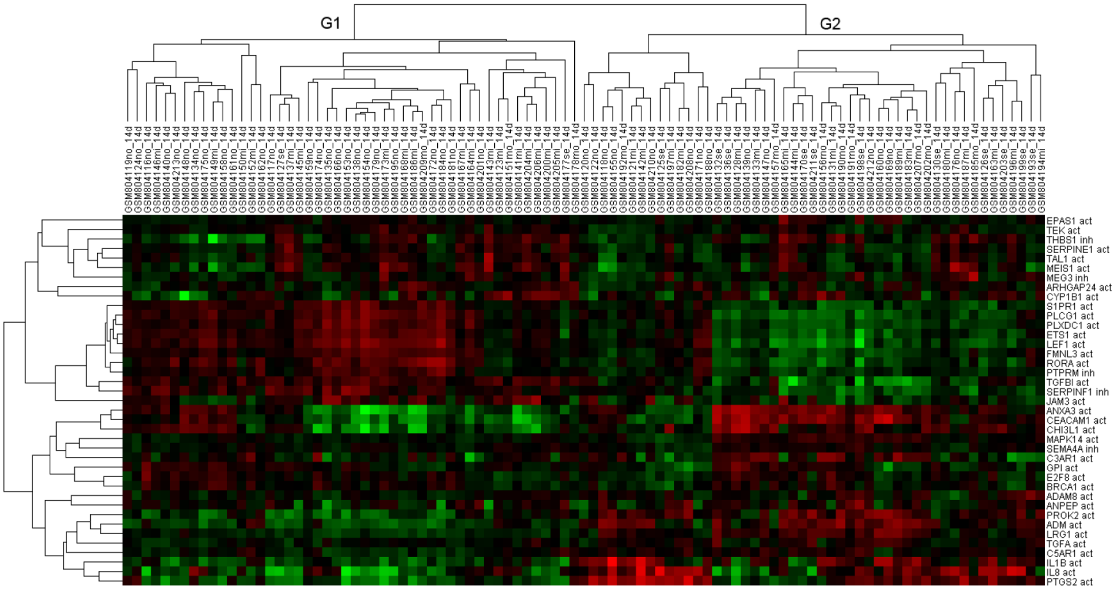

Supplementary Figure **S4**: Cluster analysis shows the 39 ARGs divide the samples of 14th day into two groups.

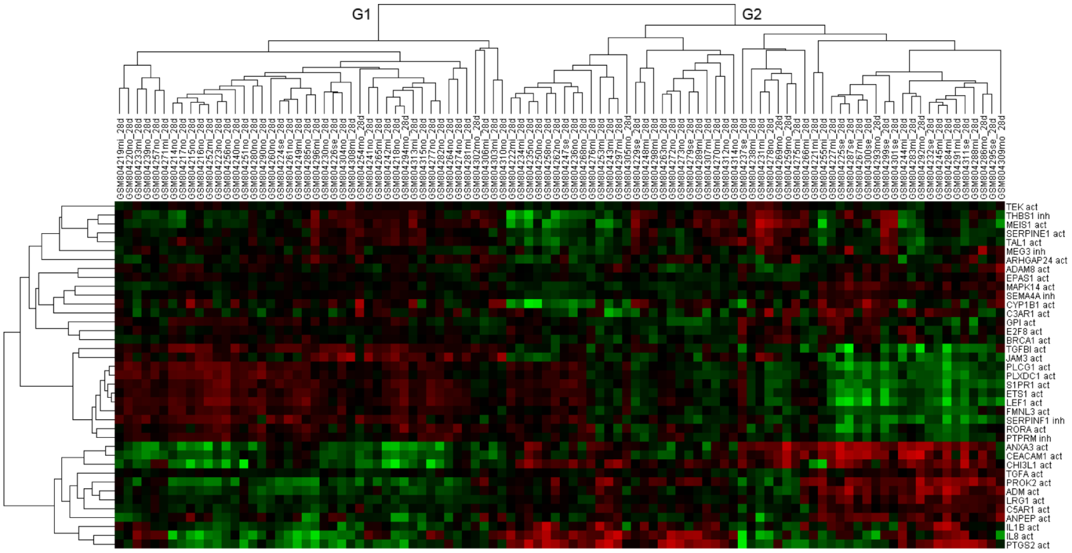

Supplementary Figure **S5**: Cluster analysis shows the 39 ARGs divide the samples of 28th day into two groups.

Supplementary **Table S3**: The proportion of BPD levels in G1 and G2 on each single day (5th, 14th, and 28th day of birth) (39 ARGs)

BPD	5day				14day				28day			
degree	G1	percent	G2	percent	G1	percent	G2	percent	G1	percent	G2	percent
no	28	60.87	7	13.73	25	52.08	14	28.57	26	59.09	12	21.43
mi	14	30.43	20	39.22	18	37.50	16	32.65	13	29.55	25	44.64
mo	2	4.35	11	21.57	3	6.25	8	16.33	3	6.82	7	12.50
se	2	4.35	13	25.49	2	4.17	11	22.45	2	4.55	12	21.43
total	46	100	51	100	48	100	49	100	44	100	56	100

no = no-BPD, mi = mild BPD, mo = moderate BPD, se = severe BPD

Supplementary **Table S4**: χ^2 test for BPD and no-BPD in G1 and G2 by 5th, 14th, and 28th day samples (39 ARGs)

	Group	no-BPD	BPD	Chi-square
5 day	G1	28	18	< 0.01
	G2	7	44	
14 day	G1	25	23	< 0.05
	G2	14	35	
28 day	G1	26	18	< 0.01
	G2	12	44	

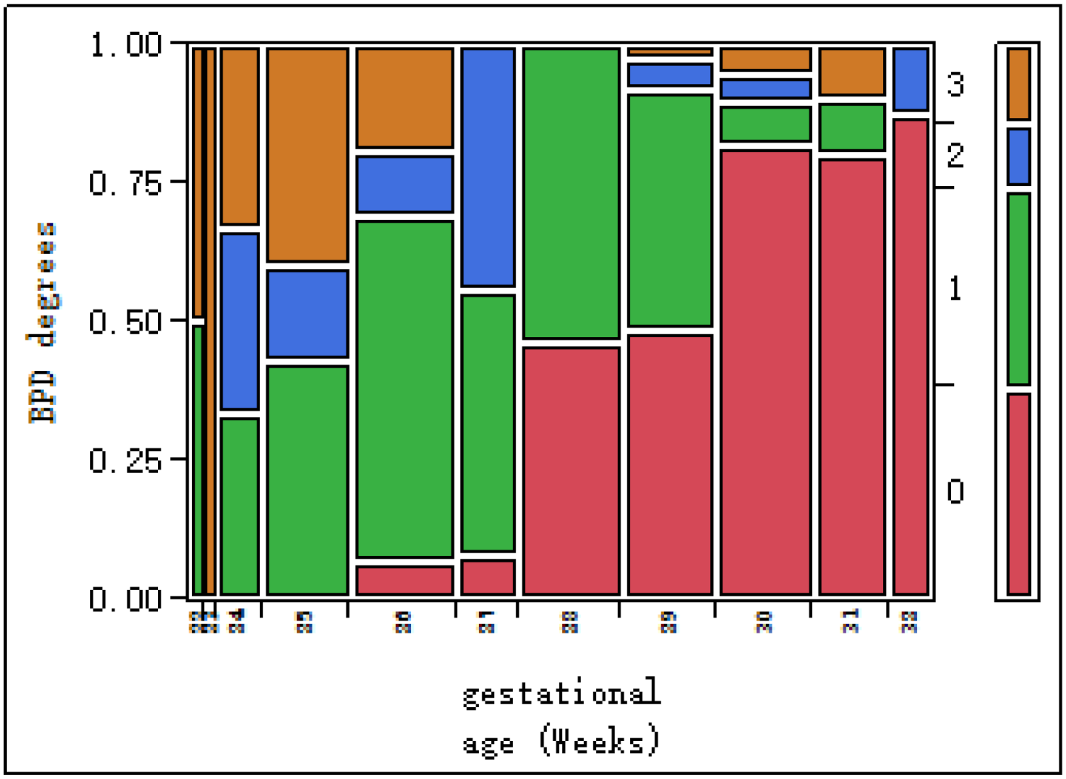

Supplementary Figure S6: Mosaic plot shows the relationship between the BPD levels and the birth gestational age. As the birth gestational age increased the no BPD portion became larger, and severe BPD portion became smaller. For BPD degree, 0 = no BPD, 1 = mild BPD, 2 = moderate BPD, 3 = severe BPD.

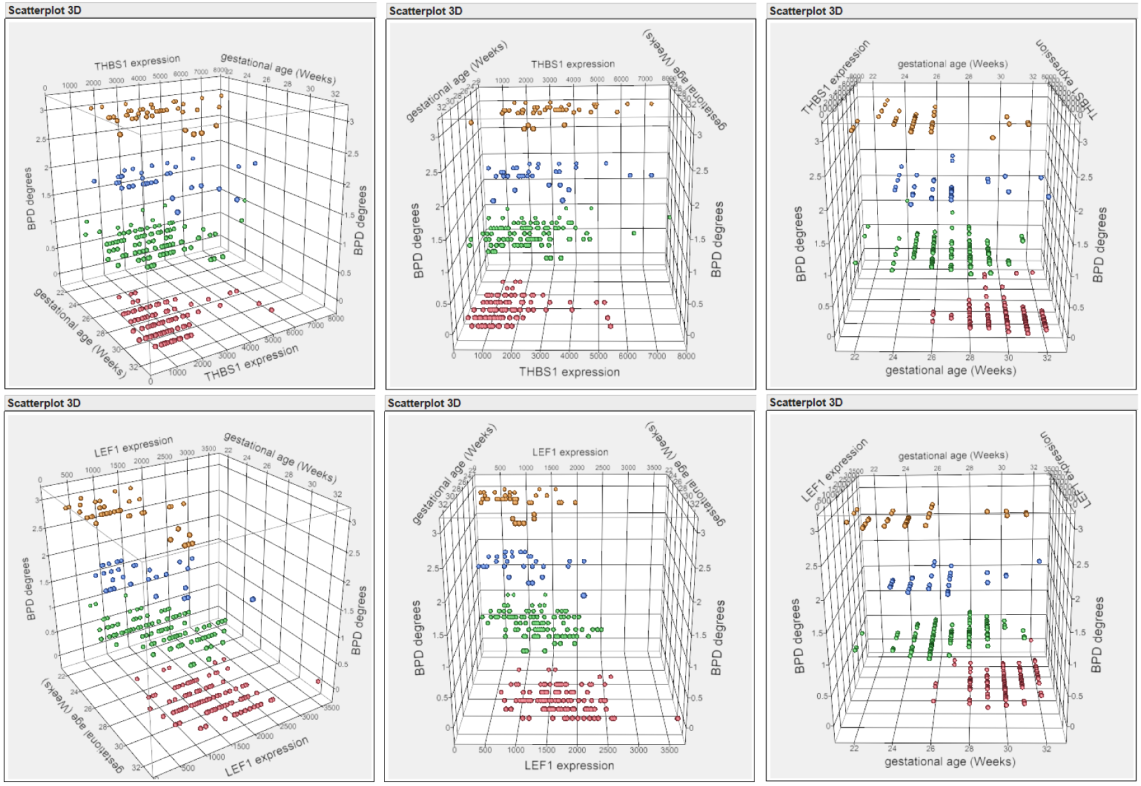

Supplementary Figure S7: 3D scatterplot shows the relationship of THBS1, LEF1 with BPD levels and gestational age respectively. The graph indicates that the younger of the infants the more serious of the disease and the higher expression of THBS1, the lower expression of LEF1.

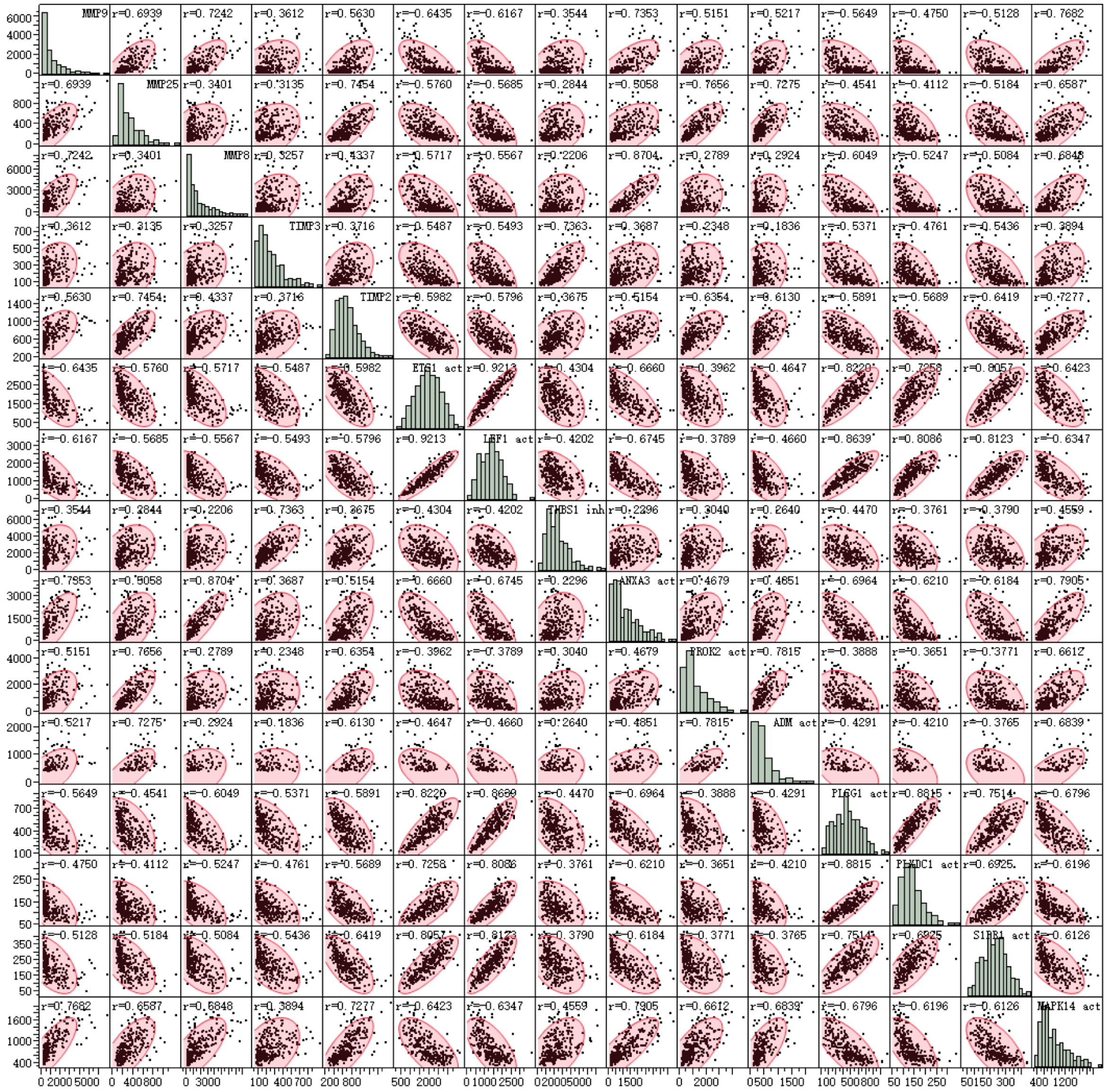

Supplementary Figure S8: Scatterplot matrix graphs show the multivariate analysis results. The correlations of the entire 39 angiogenesis-related genes and 27 MMP genes were checked, here shows the genes that we focused on in this paper. R value indicated that LEF1 and ETS1 existed good correlation ($r = 0.9213$, $P < 0.001$).

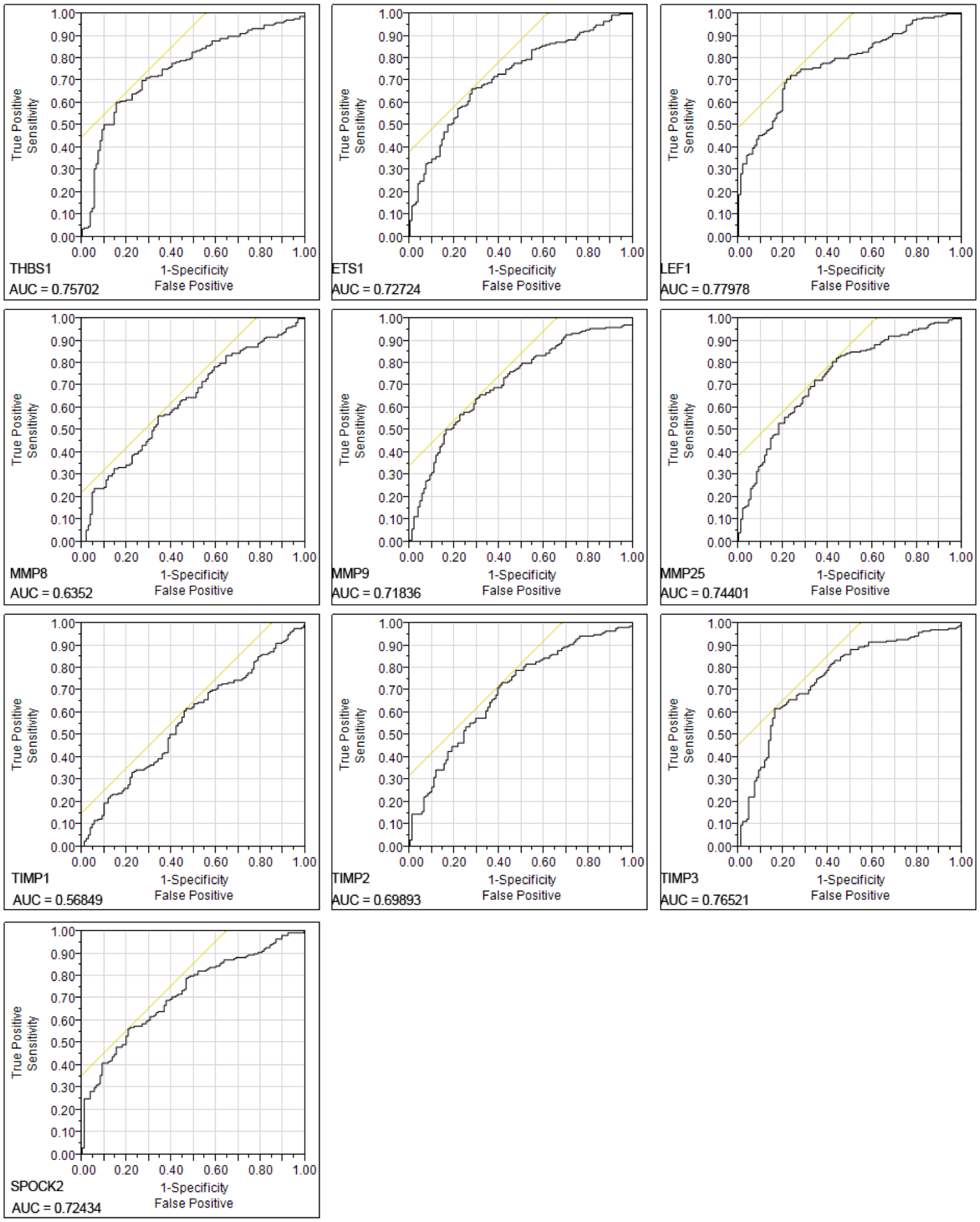

Supplementary Figure S9: Receiver operating characteristic curves (ROC) of ARGs, MMPs, TIMPs for differentiation between no-BPD and BPD samples. According to area under the curve (AUC) of all variables, ARGs had a better differential capability than MMPs and TIMPs.
